# Prediction of elevation of substantial fluctuation in coal seam floor based on surface spline function and its derivative

**DOI:** 10.1038/s41598-022-12656-w

**Published:** 2022-07-25

**Authors:** Zhenwei Yang, Junyi Xu, Zhaofeng Xu, Jiangfeng Chen

**Affiliations:** 1 Institute of Resources & Environment, Henan Polytechnic University, Jiaozuo, 454000 China; 2 Collaborative Innovation Center of Coal Work Safety and Clean High Efficiency Utilization, Jiaozuo, 454000 China

**Keywords:** Geology, Geophysics

## Abstract

With the increasing depth of coal mining, the geological stress and structure becomes more and more complex. The elevation of No.8 coal floor significantly undulates in the studied coal mine. Even though a lot of boreholes have been drilled, it remains difficult to predict the spatial distribution features and it becomes challenging to plan the tunnel for the working face. Consequently, there has been a great loss of production in the coal mine, therefore, it is of great significance to study the prediction method for coal floor elevation of the working face. The surface spline function can be regarded as an infinite flat plate deformation in pure bending. The fluctuation of the coal floor can be considered to be the result of multi-period tectonic stress applied on the coal seam, so, the prediction of elevation of the coal floor with surface spline method is feasible. An advantage of surface spline method is that any order differentiable smooth surface can be obtained without regular known lattice and boundary derivatives. In the paper, the complete expression of surface spline function is derived, which is used to predict the elevation of No.8 coal floor of the coal mine. The results show that the trend of elevation of the coal floor can be extrapolated by known data, and the maximum error is 20 m, the minimum error is 0 m, and the error of 80% of the data is less than 3 m. The general trend of the coal floor has been predicted well. However, local peaks and valleys could not be predicted correctly, therefore, the first and second order derivative are projected to predict the peaks and valleys in the head of the tunneling.

## Introduction

With the increasing mining depth, the geological condition of the coal seams is more and more complex. The plastic and rheology deformation of coal seam is common in deeply exploited coal mines^1^. The coal miners have a major problem with roadway excavation hanging over their head, because when the coal seam greatly undulates, miners can’t predict the elevation of the coal seam before excavation^2^. In this condition, many methods such as geophysical exploration methods, drilling methods and so on have been used to estimate the approximation of the geological surface. Drilling can ascertain the elevation of the coal seam floor, however, it takes too much money and time^3^. The geophysical exploration methods such as direct current resistivity, electromagnetism, seismology, etc., can also be used to predict the elevation of the coal seam floor, but the accuracy is too low to be used to practical production^4,5^.

The prediction of the distribution of the geological body underground is one of the most active fields in mathematical geology^6^. An urgent problem to be solved is predicting the fluctuation of the coal seam with existing borehole data in deeply exploited coal mines, so as to guide coal mining^7^. The spatial distribution of the coal seam is determined by the shape of its roof and floor, therefore, the prediction of the coal shape can be used to predict the surface of the coal seam floor. Such problems can be reduced to the approximate estimation of geological surfaces, therefore, the prediction problem of the coal seam floor could be solved by a numerical method^8^. The deformation of the coal seam will implicitly influence the fluid flow transport properties due to the coupled stress-permeability evolution. 3D reconstruction technology was used to simulate the deformation and water transport with geometrical coal model^9^. Fan et al. focused on the horizontal stress variation with respect to the primary depletion under uniaxial strain conditions based on a gas–solid coupling model incorporated with swelling effect. Skurtveit studied the deformation mechanism involved during shear-enhanced compaction and controlling parameters for yield stress at varying confining pressures for sandstone/sand with different grain sizes, porosities, and packing^10–12^.

The surface spline function forms a smooth geological surface by interpolating 3D discrete geological data^13,14^. The requirements of conditions for spline function are too harsh to be satisfied in coal mining practices^15^. Our purpose here is to give an example of predicting the floor undulation of the coal mine base by the surface spline method^16,17^. A vital merit of the function is that the point coordinates are not arranged regularly and the boundary conditions adopted are natural boundary conditions, and the derivate boundary condition is not needed^18,19^. The manuscript in the form of deduction of surface spline function covers the comprehensive description of the full expression and advantages for computational performance on prediction of elevation of substantial fluctuation in coal seam floor. Thus, this manuscript appears to be unique and useful for technical readers to attain a high level of comprehension on calculational approaches.

The paper is structured as follows. A background about the prediction of the distribution of the geological body based on the mathematical models and the surface spline function have been already given in Sect. “Introduction”. Once used for the interpolation calculation of aircraft surface, the mathematical principle of the surface spline function is introduced along with the full expression in Sect. “Methods”. In Sect. “The generality of the study region”, the generality of the study region and the rock mechanical property is offered. In addition, with the elevation of coal seam floor of excavated roadway, the spatial distribution of coal seam floor is introduced, and the location of unexcavated roadway is also presented in this part. In Sect. “Results and discussions”, the elevation of coal seam floor of unexcavated roadway is calculated by surface spline function. For comparing the computational accuracy, the elevation calculated by other two method is also shown. The result shows that it is an effective method to predict the elevation and tendency of the coal seam, and the first order derivative and second order derivative are also discussed to predict the peaks and valleys of coal seam. Finally, a summary of the main conclusions related to the elevation prediction of coal seam floor is given in Sect. “Conclusions”.

## Methods

### Surface spline function

Surface spline function, also known as thin plate spline interpolation function, is one of the most efficient methods to date for global interpolation of scattered data, which has been used in interpolating the calculation of aircraft surfaces^20^.

1 $$ D\nabla^{4} W = q. $$

where *D* denotes the bending strength of plate.

**Figure 1:**
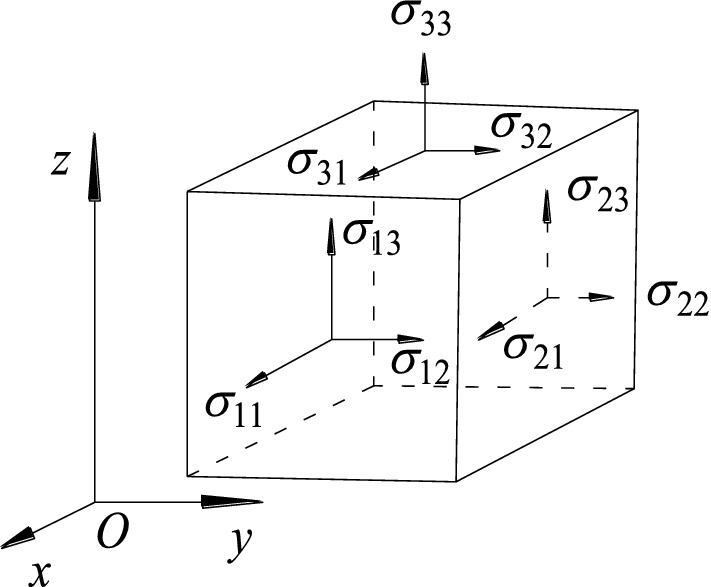
The schematic diagram of stressed cube.

The deformation of the coal mine is similar to the change of a large flat surface after stress, which is shown in Fig. 1. Let *Q*_*x*_, *Q*_*y*_ denote the stress components σ_31_ = σ_13_ and σ_32_ = σ_23_ on the veneer across the section of the axis perpendicular to *x*, *y* respectively, then

2 $$ Q_{x} = - D\frac{\partial }{\partial x}(\nabla^{2} W),\,\,Q_{y} = - D\frac{\partial }{\partial y}(\nabla^{2} W). $$

The deformation (deflection) depends on the point load *p*_*i*_ of independent *n* points (*x*_*i*_, *y*_*i*_, *i* = 1, 2,…,*n*). For calculating the deformation of a point, polar coordinates of *x* = *r*⋅cos*θ* and *y* = *r*⋅sin*θ* are introduced. Suppose the point load locates at the origin of coordinates. There is a total shear 2π*rQ*_*r*_ on the concentric circle where the radius is *r*. The value of the total shear force is equal to P and the direction is opposite, that is

3 $$ 2\pi rQ_{r} = - p. $$

Converting (2) into polar coordinate

4 $$ Q_{r} = - D\frac{d}{dr}\left( {\frac{{d^{2} W}}{{dr^{2} }} + \frac{1}{r}\frac{dW}{{dr}}} \right) = - D\frac{d}{dr}\left( {\frac{1}{r}\frac{d}{dr}r\frac{dW}{{dr}}} \right). $$

Thus

5 $$ \frac{d}{dr}\left( {\frac{1}{r}\frac{d}{dr}r\frac{dW}{{dr}}} \right) = - \frac{{Q_{r} }}{D} = \frac{p}{2\pi rD}. $$

Continuous integrating three times:

6 $$ W = \frac{p}{8\pi D}\left( {r^{2} {\text{In}}r + c_{1} r_{2} + c_{2} {\text{In}}r + c_{3} } \right). $$

In the center of the plate *r* = 0, $\frac{dW}{{dr}} = 0$, so, *c*_2_ = 0, then

7 $$ W(r) = A + Br^{2} + (p/16\pi D)r^{2} {\text{In}}r^{2} . $$

where A and B are undetermined coefficient, *p* is point load.

Deformation produced by *n* points load is superimposed by *n* solver

8 $$ W(x,y) = \sum\limits_{i = 1}^{n} {\left( {A_{i} + Bir_{i}^{2} + \left( {p_{i} /16\pi D} \right)r_{i}^{2} {\text{In}}r_{i}^{2} } \right)} . $$

where $$r_{i}^{2} = \left( {x - x_{i} } \right)^{2} + \left( {y - y_{i} } \right)^{2}$$.

Away from the points load, the surface spline will be flat or nearly flat. With the condition, some equilibrium equations can be derived. Let $x = r\cos \theta ,y = r\sin \theta$, thus

9 $$ r_{i}^{2} = r^{2} - 2r\left( {x_{i} \cos \theta + y_{i} \sin \theta } \right) + x_{i}^{2} + y_{i}^{2} . $$

10 $$ {\text{In}}r_{i}^{2} = {\text{In}}r^{2} + {\text{In}}\left( {1 - \frac{2}{r}(x_{i} \cos \theta + y_{i} \sin \theta ) + \frac{{x_{i}^{2} + y_{i}^{2} }}{{r^{2} }}} \right){.} $$

Based on series expansion

11 $$ {\text{In}}(1 - x) = - \left( {x + \frac{{x^{2} }}{x} + \frac{{x^{3} }}{3} + \cdot \cdot \cdot } \right){ (} - 1 \le x < 1{)}. $$

Get

12 $$ \begin{gathered} {\text{In}}\left( {1 - \frac{2}{r}(x_{i} \cos \theta + y_{i} \sin \theta ) + \frac{{x_{i}^{2} + y_{i}^{2} }}{{r^{2} }}} \right) \hfill \\ \;\; = - \frac{2}{r}(x_{i} \cos \theta + y_{i} \sin \theta ) + \frac{{x_{i}^{2} + y_{i}^{2} }}{{r^{2} }} + \cdots \, . \hfill \\ \end{gathered} $$

as a result

13 $$ \begin{gathered} W(r,\theta ) = r^{2} {\text{In}}r^{2} \sum\limits_{i = 1}^{n} {(p_{i} /16\pi D)} + r^{2} \sum\limits_{i = 1}^{n} {B_{i} } \hfill \\ \quad\quad\quad\quad\,\,\, - 2r{\text{In}}r^{2} \sum\limits_{i = 1}^{n} {(x_{i} \cos \theta + y_{i} \sin \theta )(p_{i} /16\pi D)} \hfill \\ \quad\quad\quad\quad\,\,\, - 2r\sum\limits_{i = 1}^{n} {(x_{i} \cos \theta + y_{i} \sin \theta )(p_{i} /16\pi D + B_{i} )} \hfill \\ \quad\quad\quad\quad\,\,\, + {\text{In}}r^{2} \sum\limits_{i = 1}^{n} {(x_{i}^{2} + y_{i}^{2} )(p_{i} /16\pi D)} + C + \cdots . \, \hfill \\ \end{gathered} $$

where *C* is constant term. The residual term *r*^-1^, *r*^-2^ etc. can be neglected when r is very large. For the quadratic term and the cubic terms, which are *r*^2^ In *r*^2^, *r*^2^ and *r*^2^ In *r*^2^, the coefficients are zero. The following equation can be obtained

14 $$ \begin{gathered} \sum {p_{i} } = 0, \hfill \\ \sum {x_{i} p_{i} } = 0, \hfill \\ \sum {y_{i} p_{i} } = 0, \hfill \\ \sum {B_{i} } = 0. \hfill \\ \end{gathered} $$

If let

15 $$ \begin{gathered} a_{0} = \sum\limits_{i = 1}^{n} {\left[ {A_{i} + B_{i} \left( {x_{i}^{2} + y_{i}^{2} } \right)} \right]} {, }\quad a_{1} = - 2\sum\limits_{i = 1}^{n} {B_{i} x_{i} } , \hfill \\ a_{2} = - 2\sum\limits_{i = 1}^{n} {B_{i} y_{i} } {, }\quad F_{1} = p_{i} /16\pi D. \, \hfill \\ \end{gathered} $$

With formula (14)–(17), formula (8) changed into

16 $$ W(x,y) = a_{0} + a_{1} x + a_{2} y + \sum\limits_{i = 1}^{n} {F_{i} r_{i}^{2} {\text{In}}r_{i}^{2} } . $$

Substituting the point load for the distributed load, and taking into account the need for higher order differentiation, the term $r_{i}^{2} {\text{In}}r_{i}^{2}$ can be substituted for $r_{i}^{2} {\text{In(}}r_{i}^{2} + \varepsilon )$, in which ε is a small amount. Then

17 $$ W(x,y) = a_{0} + a_{1} x + a_{2} y + \sum\limits_{i = 1}^{n} {F_{i} r_{i}^{2} {\text{In(}}r_{i}^{2} + \varepsilon ).} $$

*ε* is an empirical regulation parameter, which is determined according to actual condition. ε should be smaller if the curvature of the surface changes greatly, otherwise it will be a little bigger. 

Formula (18) is the complete expressions of surface spline function, where *n* + 3 unknown numbers that is *F*_*i*_(*i* = 1,2,…,*n*), *a*_0_,*a*_1_,*a*_2_. With the *n* data and formula from (10)–(12), the *n* + 3 unknown numbers could be obtained by solving the equations as follows:

18 $$ \left\{ \begin{gathered} W_{i} = a_{0} + a_{1} x_{1} + a_{2} y_{i} + \sum\limits_{i = 1}^{n} {F_{i} r_{ij}^{2} {\text{In(}}r_{ij}^{2} + \varepsilon )} \hfill \\ { (}j{ = 1,2,}...{,}n{, }r_{ij}^{2} { = (}x_{i} - x_{j} {)}^{{2}} { + (}y_{i} - y_{j} {)}^{{2}} {),} \hfill \\ \sum {F_{i} } = 0, \hfill \\ \sum {x_{i} F_{i} } = 0, \hfill \\ \sum {y_{i} F_{i} } = 0. \hfill \\ \end{gathered} \right. $$

## The generality of the study region

### Geographical location of the coal mine

**Figure 2:**
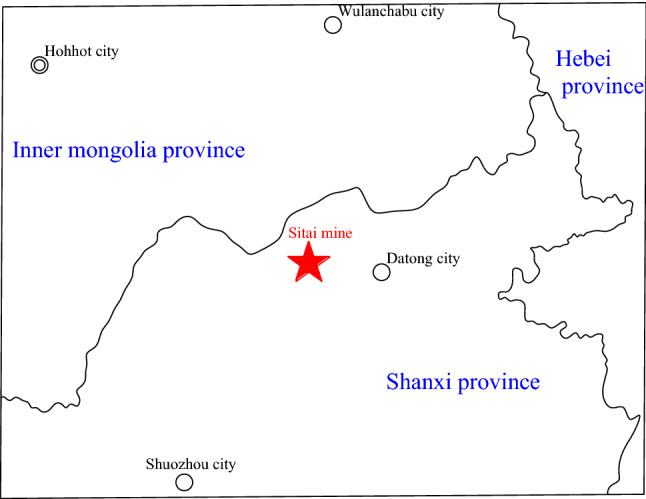
The location of the coal mine.

The coal mine that we studied lies on the northern fringe of Datong Coalfield in Shanxi province (Fig. 2), 27 km west of Datong city, which is convenient for transportation. The central geographical coordinates are 40°06′N and 113°0′. The shape of the study area, where mine working faces are higher than 890 m above sea level, is an irregular rectangle with a size 13.8 km long from south to north and 8 km wide form east to west. It is located at the north of the Shanxi plateau. The ground elevation is 1177.6 m above sea level. The terrain consists of low hilly landforms and is higher in the northwest and southeast and lower in the middle. The relative height difference is 206.5 m.

### Formation lithology

Most of the surface of the mine field is covered by loose sediments of the Quaternary. The geological stratum in the area includes upper Archean Jining group, Cambrian, Carboniferous, Jurassic and Quaternary. The Jurassic, Carboniferous and Permian strata are the main coal-bearing formations. The main lithology from old to new is mica gneiss and hornblende gneiss of upper Archean Jining group, marl and limestone of Cambrian, iron aluminum mudstone and clastic rock of Carboniferous, and sandstone of Jurassic.

There is a rock wall 3.8 km long and 10 m wide in the southeast of the coal mine, which was uncovered during mining of the Jurassic coal seam. The structure is granular and stomatal. The metamorphism range of the coal seams on both sides is less than 1 m. It has little effect on coal seam mining^21^.

### Geological structure

The research area is on the east side of the Luliang meridional structural belt. To the north it is connected to the Tianshan ~ Yinshan tectonic belt. It is the joint part of the east–west tectonic system and the New China Department. The coal mine lies in the north of Datong coalfield. The geological structure is very complicated, and is known as the ‘geological museum’ of Datong coalfield. The general strike is NE ~ SW. The collapse column is developed. There is intrusion of magmatic rocks. The geological structure type of the coal mine is medium^22,23^.

**Figure 3:**
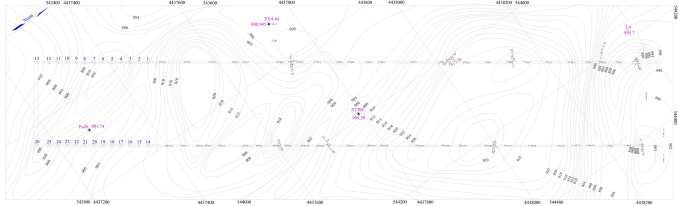
Contour of coal seam floor of 80,207 working face (plotted by borehole data of the minefield).

**Figure 4:**
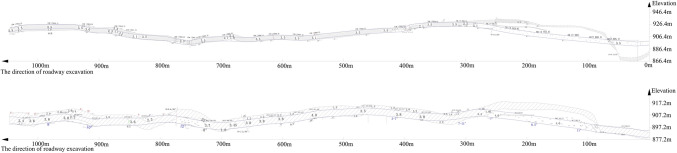
The elevation of coal seam floor of haulage roadway and air-return roadway (the blue line is the roof and bottom of the roadway, the shaded area is the roof and the floor of the coal seam).

There are four normal faults in the coal mine, which is fault *F*_1_, fault *F*_2_, fault *F*_3_ and fault *F*_4_. The fault *F*_1_ lies in the southwest with 2.1 km in length in the mine, extends NW, dips NW, and the maximum drop is 27 m. The fault *F*_2_ lies in the southwest with 1.6 km in length in the mine, extends NE, dips SE, and the maximum drop is 11 m. The fault *F*_3_ lies in the southwest with 2.2 km in length in the mine, extends NEE, dips NW, and the maximum drop is 16 m. The fault *F*_4_ lies in the north with 0.55 km in length in the mine, extends NE, dips NW, and the maximum drop is 10 m. As is shown in the Figs. 3 and 4.

### The coal-bearing strata and rock mechanical property of No.8 coal seam

The thickness of the coal-bearing strata is 0–8.10 m, average 3.52 m. The hardness of No.8 coal floor is semi-hard to weak. The false roof appears sporadic and the immediate roof distributes in almost the whole area. The main roof distributes all of the area except northwest and part of southeast area^24^. The data on the rock mechanical property of No.8 coal seam is shown in the Table 1.

**Table 1:** The rock mechanics testing results of immediate roof for No. 8 seam.

Drilling number	Lithology of Immediate roof	Compressive strength (MPa)		Softening coefficient (MPa)	Tensile strength (MPa)	Shear strength (MPa)
		Natural state	Saturation state			
ST302	Coarse sandstone	6.2 ~ 18.4	10.3 ~ 15.9	0.95	0.53 ~ 1.43	1.34 ~ 1.78
	Siltstone	19.4 ~ 28.4	19.0 ~ 30.3	0.97	1.13 ~ 1.71	4.85 ~ 8.41
ST306	Fine-sandstone	17.5 ~ 30.4	11.0 ~ 28.9	0.87	0.65 ~ 0.81	1.73 ~ 2.27
	Sandy mudstone	12.0 ~ 22.2	15.3 ~ 20.0	0.99	1.09 ~ 1.21	3.06 ~ 4.03
ST703	Mudstone	27.6 ~ 33.7	11.2 ~ 23.4	0.53	1.14 ~ 1.16	3.06 ~ 5.21
	Coarse sandstone	10.0 ~ 49.6	11.2 ~ 15.8	0.36 ~ 0.71	0.82 ~ 3.32	1.73 ~ 3.23
ST708	Fine-sandstone	19.5 ~ 35.9	16.1 ~ 22.7	0.67	1.51 ~ 1.84	4.13 ~ 4.33
	Coarse sandstone	13.3 ~ 25.5	10.7 ~ 23.8	0.88	0.82 ~ 0.93	2.56 ~ 4.03
	Sandy mudstone	23.2 ~ 37.3	19.7 ~ 32.2	0.88	0.58 ~ 1.38	2.41 ~ 2.93
	Mudstone	18.2 ~ 25.1	9.1 ~ 24.1	0.74	0.42 ~ 0.72	3.09 ~ 4.08

## Results and discussions

### Results

With boreholes (ST304, Pu26) and roadways, the elevation data of the coal floor reveals that the tunneling direction has been predicted based on surface spline method, which is shown in Table 2. From Table 2, we can see that the maximum error of predictive elevation value of the haulage roadway and air-return roadway is 20 m, and the minimum error is 0 m. Compared to the exposed elevation, the errors of 80% predictive elevation is less than 3 m. The trend of the coal floor has been predicted well.

**Table 2:** Exposed actual value and predictive elevation calculated based on surface spline method.

Point number	Predictive elevation (m)	Exposed actual elevation (m)	Point number	Predictive elevation (m)	Exposed actual elevation (m)
1	915.7	906.0	14	906.6	908.6
2	913.1	909.2	15	905.4	907
3	910.3	906.8	16	904.8	906.9
4	907.6	907.6	17	903.7	905.5
5	904.9	907.7	18	902.6	904.2
6	902.2	905.7	19	901.4	906.4
7	900.0	895.4	20	900.5	908
8	897.3	893.9	21	899.7	906.9
9	894.9	894.2	22	898.8	904.4
10	892.8	894.9	23	898.1	903.7
11	890.6	895.0	24	897.5	903.3
12	888.6	900.0	25	896.7	898.6
13	886.1	903.9	26	895.7	895.5

**Figure 5:**
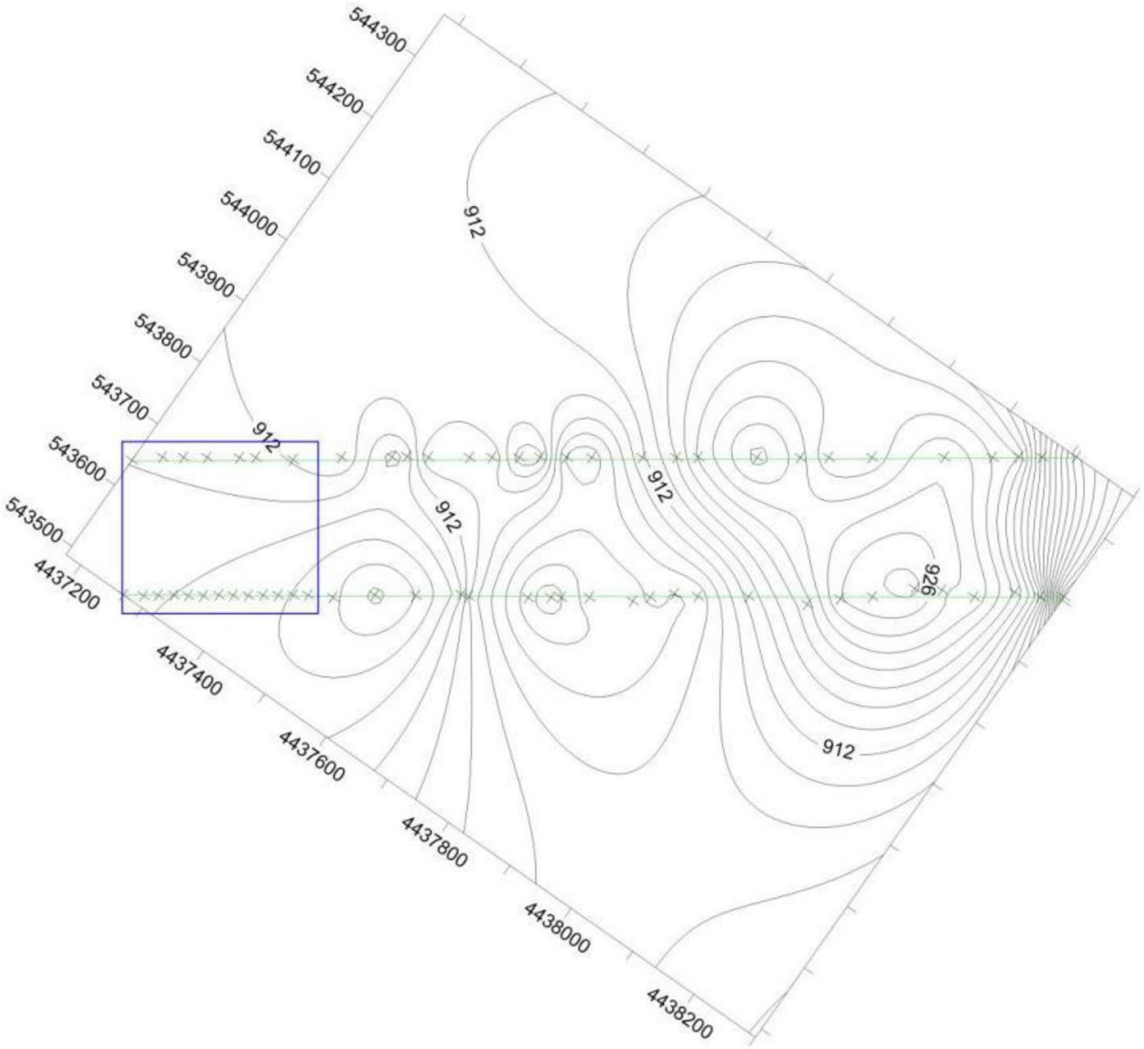
The elevation of points that enclosed by blue rectangle are calculated by Kriging method.

**Figure 6:**
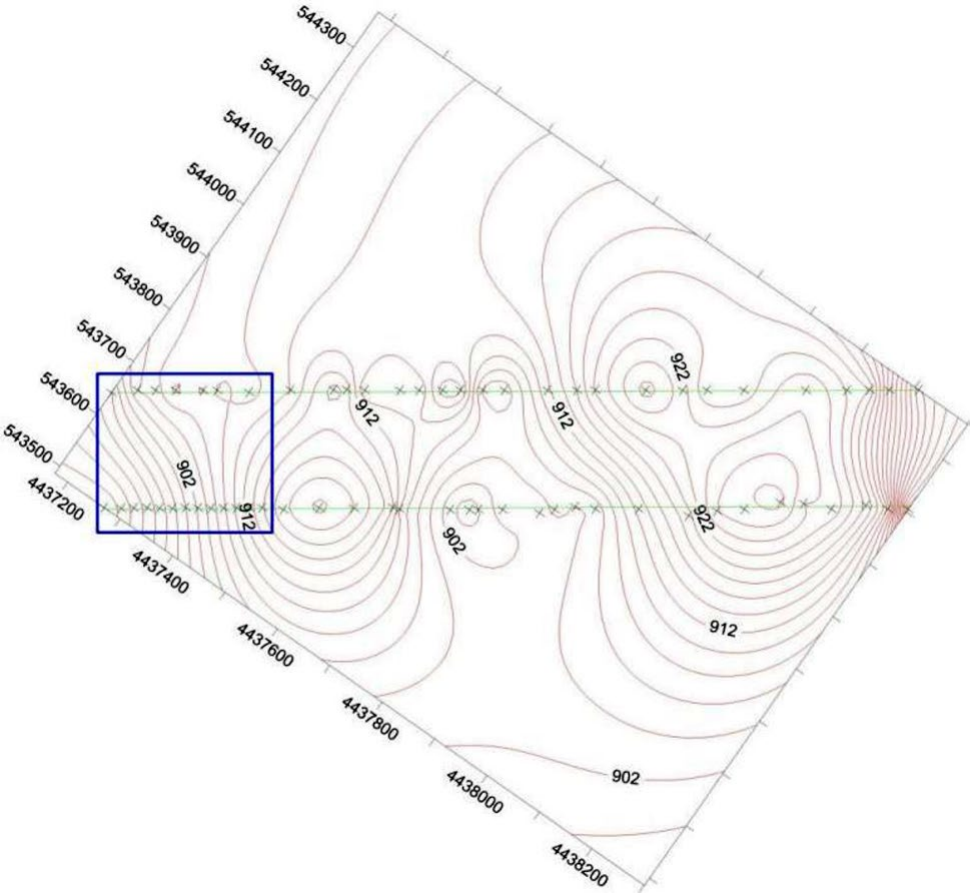
The elevation of points that enclosed by blue rectangle are calculated by surface spline function.

The elevation of the unexplored area can also be calculated by traditional interpolation methods, such as kriging, inverse distance weighted, spline, discrete smooth interpolation, etc. These methods are either based on principles of statistics or weighted average etc. The surface spline function is deducted from thin plate spline interpolation, which is good at simulating deformation naturally. Therefore, compared to surface spline function, the precision of traditional interpolation methods is not high enough. We have predicted the elevation of the coal floor with data based on 2 boreholes and 50 elevations exposed by drivage with kriging method and surface spline function. The contour of elevation of the coal floor is predicted by kriging method and surface spline function, which is shown as Figs. 5 and 6. In Figs. 5 and 6, the two straight lines represents the haulage roadway and air-return roadway. The symbol × out of the blue rectangle represents the elevation point exposed by roadways, and the symbol × in the blue rectangle represents the elevation point predicted by Kringing method (Fig. 5) and surface spline function (Fig. 6).

**Figure 7:**
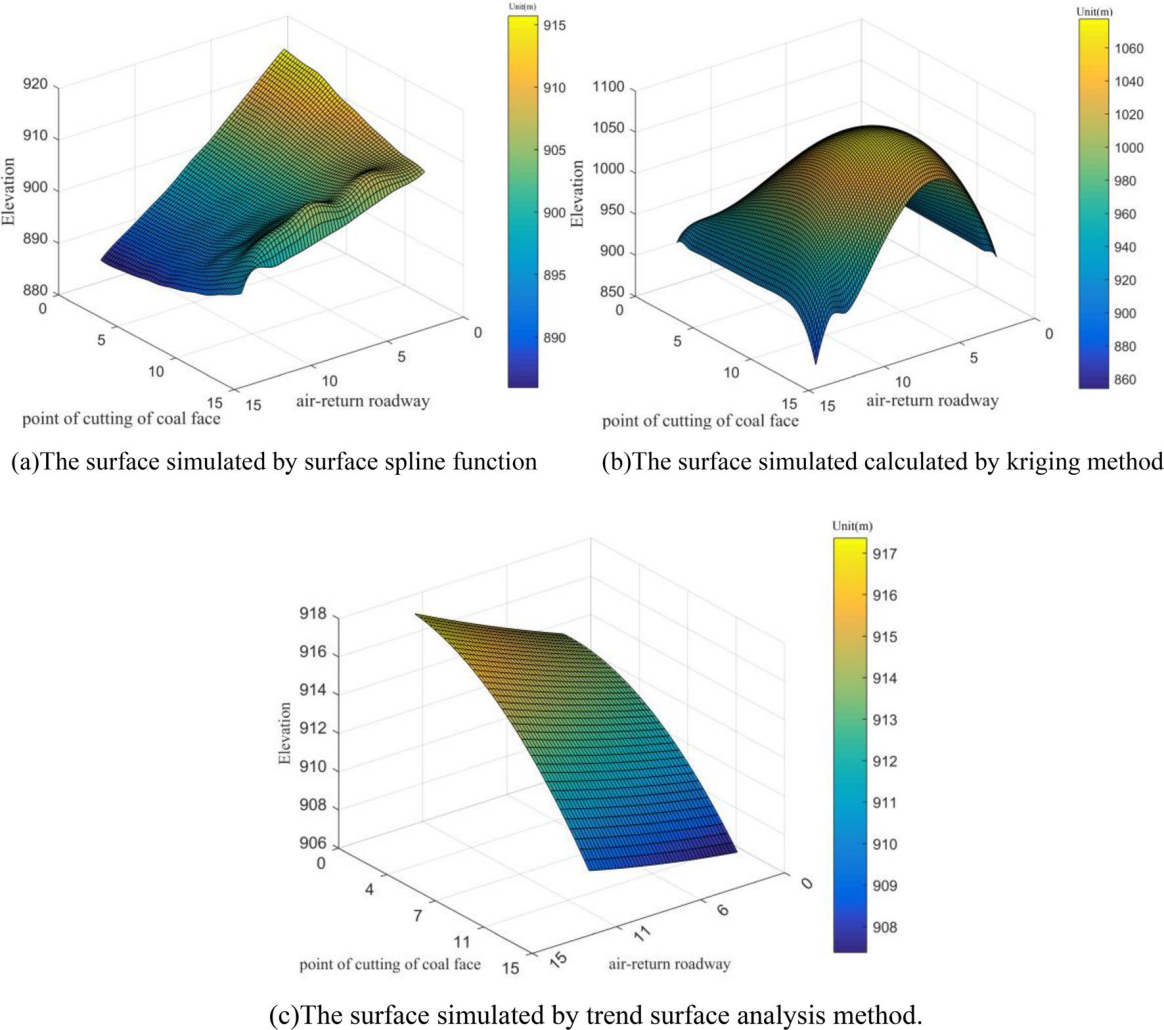
The spatial distribution of coal seam floor (enclosed by blue rectangle in Fig. 6) predicted by surface spline function, kriging method and trend surface analysis method.

In order to comparing the computational accuracy intuitively, the spatial distribution of surface of the coal seam floor(enclosed by blue rectangle in Fig. 6) is also simulated by surface spline function, kriging method and trend surface analysis (Fig. 7). According to Fig. 7, the surface spline function leads to a better match compared to the other two methods, which simulates the fluctuation of coal seam floor realistically. In Fig. 7b, because the kriging method is mainly used for interpolation, the three boreholes data around the working face is added into the surface construction. Thus, there is a big error in the middle part of the surface. In Fig. 7c, the main trendency of the coal seam floor has been predicted, but the strike deviates from the direction of regional structure. Furthermore, the surface is too smooth to be used for characterizing the coal seam floor.

**Figure 8:**
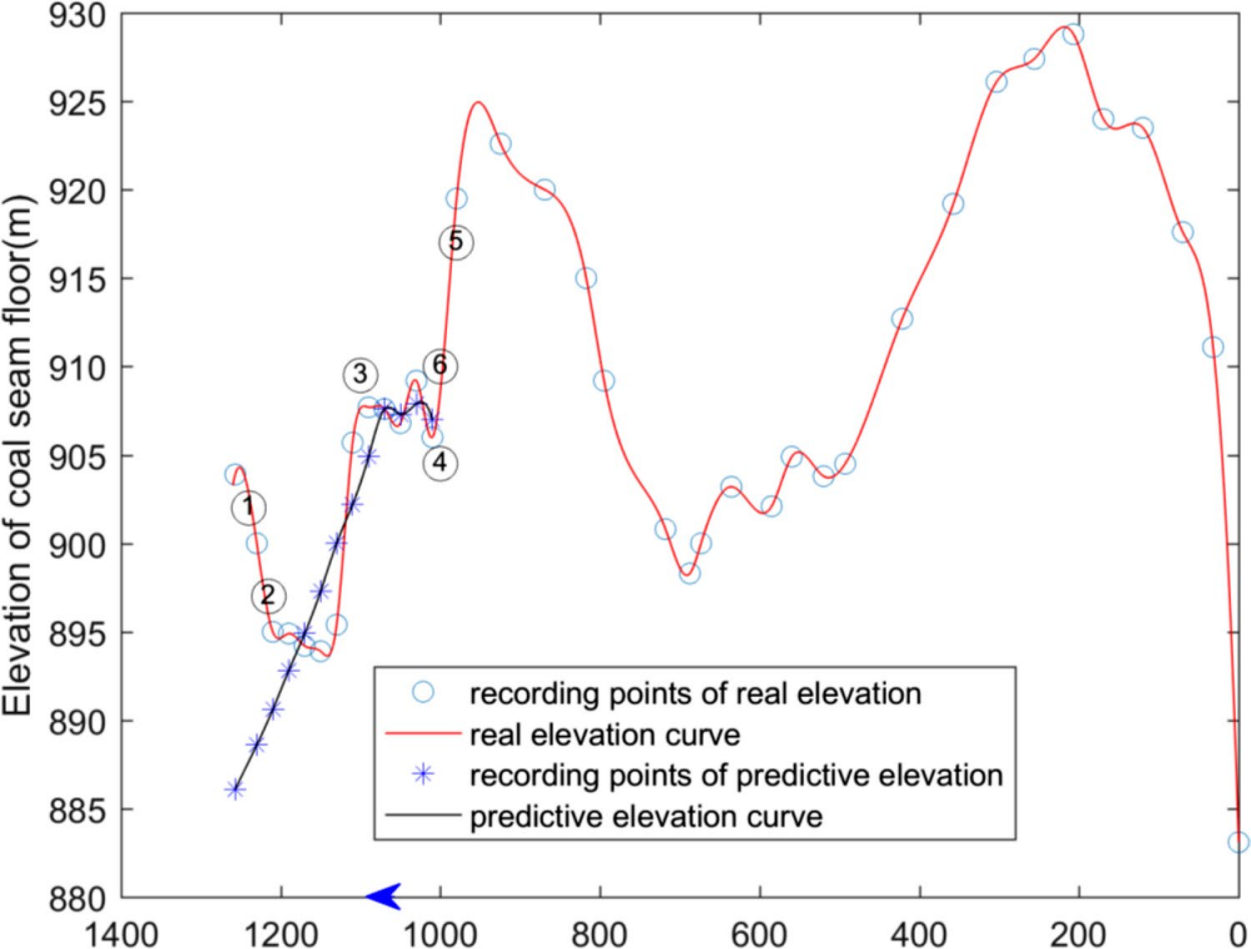
Curve of coal seam floor of haulage roadway (The blue * represents the elevation point calculated by surface spline function. The blue ◦ represents the real elevation point exposed by drivage. The arrow represents the direction of laneway excavation. The symbol ① ~ ⑥are interpreted in the Table 3).

**Figure 9:**
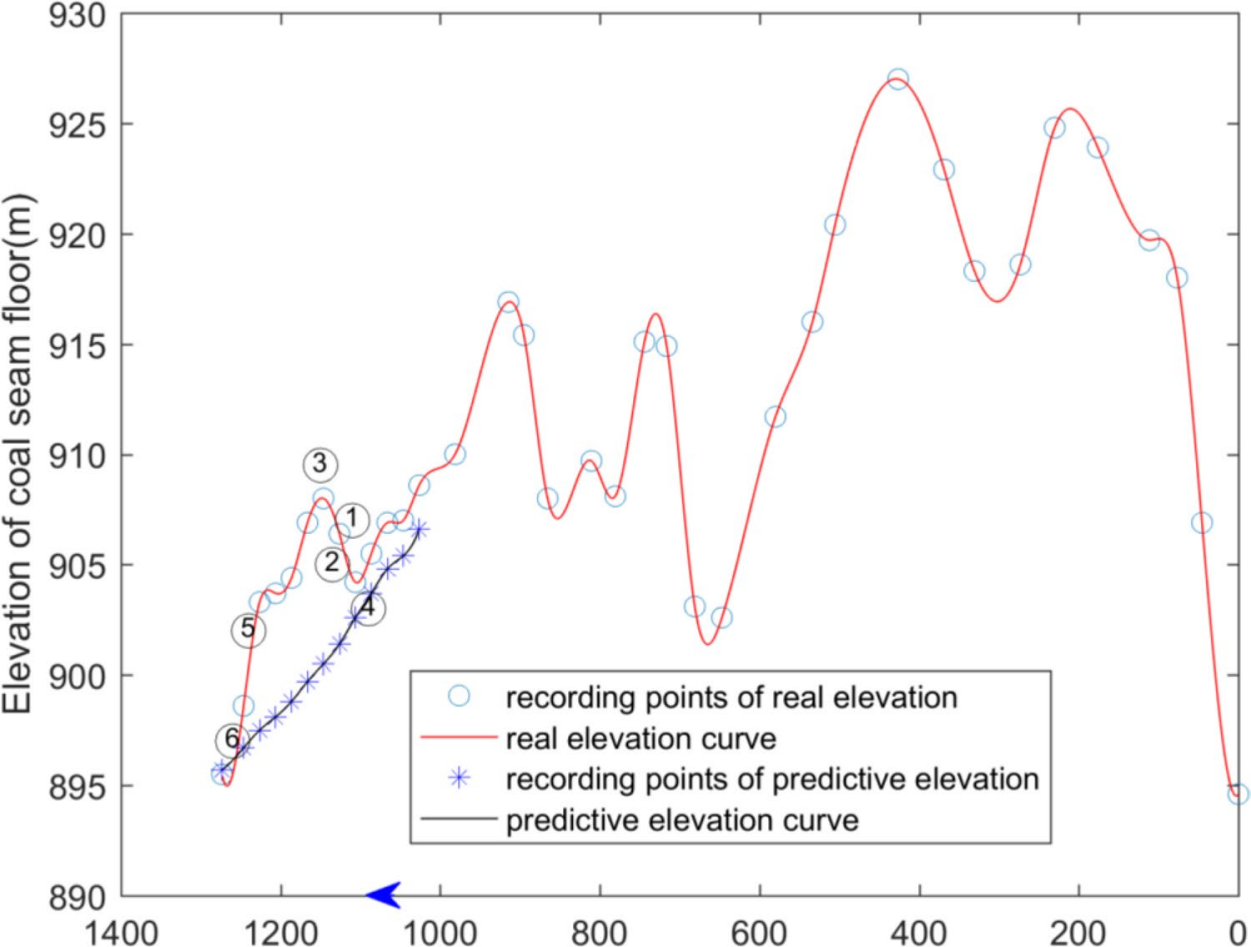
Curve of coal seam floor of air-return roadway (The blue * represents the elevation point calculated by surface spline function. The blue ◦ represents the real elevation point exposed by drivage. The arrow represents the direction of laneway excavation. The symbol ① ~ ⑥are interpreted in the Table 3).

The elevation of the coal seam floor exposed by drivage and predicted by surface spline function are also drawn in the form of 2D curve, which is shown in Figs. 8 and 9. The horizontal axis represents the roadway and the coordinate value represents the distance along the heading direction of the roadway. The vertical axis represents the floor elevation of the coal seam. The blue star represents the exposed floor elevation of the coal seam in the coal lane. The red circle indicates the elevation data of the exposed seam floor. The blue curve is the fitting curve of the seam floor elevation. The red curve is the fitting curve for predicting the elevation of the coal seam floor.

It can be clearly seen from the two figures that the trend of elevation prediction of the coal seam floor is in compliance with the trend of elevation exposed by drivage. As the distance from the heading faces area increases, the errors of predicted elevation became higher, that is, there is a higher reliability near the known area. However, with the distance increasing, the precision of prediction of the coal seam floor becomes lower.

### Discussions

With the surface spline function, the elevation information for the coal seam floor in an unknown area is analyzed, however, there is still a problem with the prediction. In the process of roadway tunneling, it is impossible to accurately predict the angle of roadway tunneling, therefore, the accuracy of elevation of the coal seam floor in front of the roadway excavation needs to be improved. Considering the continuity and gradual change of undulation of the coal floor, the undulating characteristics of the coal seam floor in front of roadway excavation were judged by the first and second derivatives of the coal seam floor in the direction of roadway excavation.

Predicting the change in the coal seam floor by surface spline function requires takes the excavation head as the reference point, and takes the heading direction of the roadway as the *x-*axis, and takes the vertical direction as the *y*-axis. The dip angle is greater than 0, which means that the space of coal seam floor is inclined and upward distributed in front of roadway excavation. The dip angle is less than 0, which means that the space of the coal seam floor in front of the roadway is inclined downward. The method to predict dip change of the coal seam by first and second derivatives at the heading head is shown in Table 3. For example, the first derivative of floor elevation of the coal seam in front of tunneling is equal to 0, and the second derivative is greater than 0, a local maximum elevation of the coal seam floor has been reached. The elevation of the coal seam floor in front of roadway excavation is about to decrease. The conditions are shown in Figs. 8 and 9.

**Table 3:** The suggestion for drifiting based on derivative value.

Conditions	The first derivative(FD)	The second derivative(SD)	Suggestion for drifiting
①	FD < 0	SD > 0	Increasing downward dip
②	FD < 0	SD < 0	Decreasing downward dip
③	FD = 0	SD > 0	Changing drifiting vertically(upward)
④	FD = 0	SD < 0	Changing drifiting vertically(downward)
⑤	FD > 0	SD > 0	Increasing upward dip
⑥	FD > 0	SD < 0	Decreasing upward dip

## Conclusions

The main conclusions can be summarized as follows:

1. The coal seam floor is regarded as a pure bending deformation surface of an infinite flat plate. Based on elasticity mechanics, the spline function approximating the surface of the coal seam floor has been deduced. Compared to common geological surface interpolating algorithm, the surface spline function fits the spatial distribution of the coal seam floor surface well. It plays an important role in guiding the tunneling of the coal face where the coal floor seam fluctuates greatly.
2. With the existing elevation data of the coal seam floor (4 drilling data and 46 elevation data exposed by roadway drivage), the surface spline function is applied to predict the tendency of coal seam elevation of a mine in North China. The results show that the trend of elevation of the coal floor can be extrapolated by known data, and the maximum error is 20 m, the minimum error is 0 m, the error of 80% data is less than 3 m. On the whole, the spatial distribution of the coal seam floor at the work face can be predicted well, however, the prediction accuracy is poor where the coal seam undulation changes dramatically.
3. The spline surface function is used to predict the floor elevation of the coal seam in front of the tunneling roadway. However, the latest exposed elevation data of the coal seam floor are not fully used for prediction in roadway drivage. Therefore, based on the variation characteristics of the first and second derivatives of the coal seam floor, which indicate the spatial variation direction of the coal seam floor in the front of the excavation it can further provide technical support for the roadway excavation in a coal mine with severe fluctuation in the coal seam.

## Data Availability

The data used to support the findings of this study are available from the corresponding authors, upon reasonable request.
